# High adherence to intermittent and continuous use of a contraceptive vaginal ring among women in a randomized controlled trial in Kigali, Rwanda

**DOI:** 10.3389/fgwh.2024.1278981

**Published:** 2024-04-11

**Authors:** Evelyne Kestelyn, Jennifer Ilo Van Nuil, Marie Michelle Umulisa, Grace Umutoni, Alice Uwingabire, Irith De Baetselier, Mireille Uwineza, Stephen Agaba, Tania Crucitti, Thérèse Delvaux, Janneke H. H. M. van de Wijgert

**Affiliations:** ^1^ Rinda Ubuzima, Kigali, Rwanda; ^2^ Oxford University Clinical Research Unit, Hospital for Tropical Diseases, Ho Chi Minh City, Vietnam; ^3^ Nuffield Department of Medicine, Centre for Tropical Medicine and Global Health, University of Oxford, Oxford, United Kingdom; ^4^ Department of Clinical Sciences, Institute of Tropical Medicine, Antwerp, Belgium; ^5^ Experimental Bacteriology Unit, Institut Pasteur Madagascar, Antananarivo, Madagascar; ^6^ Department of Public Health, Institute of Tropical Medicine, Antwerp, Belgium; ^7^ Julius Center for Health Sciences and Primary Care, University Medical Center Utrecht, Utrecht University, Utrecht, Netherlands

**Keywords:** NuvaRing, contraceptive vaginal ring, adherence, mixed methods, Rwanda

## Abstract

**Background:**

The introduction of female-initiated drug-delivery methods, including vaginal rings, have proven to be a promising avenue to address sexually transmitted infections and unintended pregnancies, which disproportionally affects women and girls in sub-Saharan Africa. Efficient uptake of existing and new technologies such as vaginal rings requires in depth understanding of product adherence. This remains a major challenge as data on adherence to vaginal rings from African countries is limited. In this study, we explored adherence of contraceptive vaginal ring (NuvaRing®) use in Kigali, Rwanda using a mixed methods approach.

**Methods:**

We collected quantitative and qualitative data at multiple time points from women participating in a clinical trial exploring the safety and acceptability of either intermittent or continuous use of the NuvaRing®. Various adherence categories were used including monthly and cumulative adherence measurement. The quantitative data were analysed using R and the qualitative data were analysed using a deductive, content-analytical approach based on categories related to the quantitative adherence measures. All data were compared and triangulated.

**Results:**

Data from 120 enrolled participants showed that self-reported adherence was high at every study visit in both study groups. At first study visit 80% of the intermittent ring users and 79.7% of the continuous ring users reported perfect adherence (assessed as “the ring was never out”). Reporting of ring expulsions and removals were highest (28.3%) at the beginning of the trial. Self-reported perfect ring adherence increased during the study and reports of ring expulsions and removals declined as familiarity with this contraceptive method increased. The percentage of women with perfect cumulative adherence was non-significantly higher in the intermittent (61.7%) than in the continuous use group (54.3%). The low rate of discrepant adherence data after triangulation (6%) is in line with the perception of the participants as adherent throughout the study.

**Conclusions:**

Self-reported adherence in both study groups was high with removals and expulsions being within the expected product range. Comprehensive adherence data triangulation allowed for a deeper understanding of context-driven behaviour that shaped adherence patterns and challenges. Our data categorisation and triangulation approach has shown potential for implementation in future vaginal ring studies aiming to better understand and measure adherence.

## Introduction

The burden of sexually transmitted infections (STIs), including HIV, and unintended pregnancies in women living in low- and middle-income countries remains high as was highlighted by the latest UNAIDS report “In Danger” (1). In 2021, women and girls in sub-Saharan Africa accounted for 63% of all new HIV infections and had the highest proportion of unmet need (21%) for modern contraception (2, 3). Women living with HIV, or in areas with high HIV prevalence, also have higher unmet needs for family planning and reproductive health services compared to the general population (4). Published data has shown that with an increase in family planning choices, there has been an increase in contraceptive users and, the reproductive health product pipeline of female-initiated drug-delivery methods, including vaginal rings, has progressed rapidly in recent years (5–9). Some examples of newly approved methods are a long acting (12-month) segesterone acetate/ethinyl estradiol contraceptive vaginal ring, the progesterone ring Progering® for postpartum women, and vaginal rings for HIV prevention such as the monthly dapivirine ring (10–13).

Whilst increasing choice remains crucial, efficient uptake of existing and new vaginal rings requires an in-depth understanding of user preferences, and barriers and facilitators to acceptability and adherence (8, 14–20). Multipurpose prevention technologies (MPTs) targeting combination(s) of HIV, other STIs, and unintended pregnancy are currently under development. MPTs, and especially long-acting MPTs, could be a game changer for many women and may improve adherence (5, 8, 21–23). For example, studies have shown that many women perceive their risk of getting pregnant as higher than their risk of getting infected with HIV (16, 24). In addition, contraceptive use is more normalized in most countries, and less controversial to discuss, than HIV prevention use (24). However, MPTs may also present new challenges; vaginal ring use for HIV prevention has to be continuous whereas vaginal ring use for contraception has historically been intermittent with a one week break to allow for menstruation (18, 21, 23–26).

Product adherence remains a major challenge (27). The female-initiated drug-delivery research field has seen an increase in user preferences research and a broader interest in socio-, cultural- and economic factors including partner and peer influences, but research into better (qualitative) adherence measures has been limited; most researchers still rely solely on self-report whereas others have focussed on potential biomarkers (28–33). A clinical trial was conducted among Rwandan women to explore the safety and acceptability of a contraceptive vaginal ring (NuvaRing®). Women were randomized to either intermittent use or continuous use of this ring. The trial results showed that the use of the contraceptive vaginal ring was safe and reported a high acceptability in both groups (19, 34,35). However acceptability and adherence to a product are intrinsically linked and there is limited evidence on adherence to vaginal rings including contraceptive vaginal rings (28–33). In this study we aimed to better understand self-reported adherence patterns in intermittent and continuous first-time vaginal ring users to inform the future introduction of HIV prevention or MPT vaginal rings that require continuous use.

## Methods

### Study design

The RingPlus study was an open-label single-centre clinical trial with randomisation to either an arm of intermittent or an arm of continuous NuvaRing® use to evaluate its safety among Rwandan women (clinicaltrials.gov registration NCT01796613). The trial was conducted from June 2013 to March 2014 at the Rinda Ubuzima research site in Kigali, Rwanda, and included a comprehensive social science component to explore ring use acceptability and adherence. The study protocol, the primary safety outcomes, and the primary acceptability outcomes have been published elsewhere (19, 34–36). However, key methods are also summarized here to facilitate interpretation of the results.

### Study participants

In order for women to be eligible for the study, they had to be in good physical and mental health, HIV negative, sexually active, and between 18 and 35 years old. They should not currently be using modern contraceptive methods but should be interested in, and medically eligible for, initiating hormonal contraceptive use. Exclusion criteria included currently smoking or breastfeeding, current use of antimicrobial medication, or having any medical contraindications to NuvaRing® use. All participants provided written informed consent.

### Sample size

This clinical study sample size calculation was based on the primary objective to assess the pre-post changes in the vaginal microbiome. More details can be found in the protol publication (34). For the qualitative research, sample sizes was determined by when data saturation was reached.

### Study procedures

Due to the differences in ring use (intermittent or continuous use), this study was an open-lable study. At enrolment, women were randomly assigned to either intermittent or continuous use of the ring based on the clinical trial database generated allocation codes. Treatment allocation was concealed until a participant has provided informed consent, was confirmed eligible, was included in the study and had inserted the CVR (34). The participants were taught how to use the ring and asked to insert their first ring in the presence of a female research nurse. If the participants felt uncomfortable for the initial insertion, a nurse or physician would help with insertion. Participants were advised to keep the ring in during menses and daily activities. They were counselled and received written instructions on how to clean and reinsert the ring in case of accidental expulsions or after purposeful removals. The total follow-up duration was a maximum of 14 weeks with a maximum of seven study visits. Intermittent users wore three rings, and continuous users wore four rings, during the study. Scheduled follow-up visits coincided with times of ring removal and/or insertion. At all ring removal visits, a physical and pelvic examination was done which included verification of the ring *in situ*, samples were collected, and case report forms (CRFs) and an interviewer-administered questionnaire (IAQ) were completed. Risk-reduction counselling and testing, condoms and treatment for curable STIs were offered as well as referrals for other medical conditions.

### Data collection

**Figure 1:**
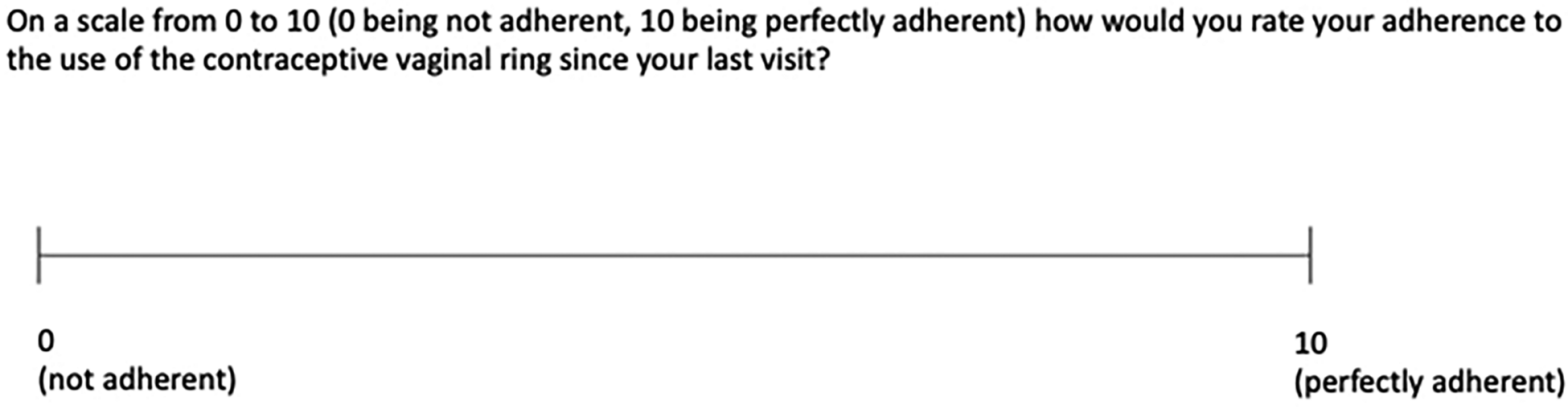
The horizontal visual analogue scale (VAS) used to assess self-reported adherence.

The team used a mixed methods approach, including triangulation of quantitative and qualitative data sources on adherence during and after the study (37). Study staff administered CRFs and IAQs at enrolment before the first ring insertion and at every ring removal follow-up visit. The CRFs documented the physical and pelvic examination findings, laboratory test results, social harms, adverse events and/or concomitant medications, and data on ring use and adherence. The latter was based on a discussion with the participant using a diary card that she had completed at home [Supplementary Appendix 1: Diary card]. Diary cards were provided at each visit to document all ring removals, expulsions, as well as sexual acts and vaginal practices between visits. The IAQs included (open-ended) questions on sexual activities, menses, ring acceptability and adherence (38). During the IAQ, the team collected qualitative data to better understand the participants’ underlying behaviours and what influenced them during the study. Participants were also asked if the ring ever came out of the vagina since their last visit, with detailed follow-up questions regarding duration of and reason for each ring removal and/or expulsion, and whether the ring was cleaned and reinserted. This detailed questioning was to allow the study team to evaluate whether more expulsions and/or removals would lead to reduced adherence as mentioned in other publications (39) Participants were also asked to complete a visual self-rating adherence scale (ranging from 0% to 100%) at each study visit (Figure 1).

The team collected qualitative data to better understand the participants’ underlying behaviours and what influenced them during the study. In-Depth Interviews (IDIs) with purposively selected study participants were conducted during the study and after study completion until data saturation was reached. This comprehensive data collection approach was complemented by triangulation of CRF, IAQ, and diary data at regular intervals during the study, documenting any discrepancies on comparison forms. At the last study visit, study nurses discussed all inconsistencies documented on the comparison forms with the participants using individually tailored open-ended questions. At that same last visit, anonymous ballot box questionnaires were collected for all participants. These questionnaires consisted of six questions, which included (among other things) questions about overall adherence and women's satisfaction with study participation. Data triangulation was planned in two phases based on a methodology by Pool et al. (37). Phase one consisted of comparing individual participant data from different data collection tools and clarifying discrepant results with those participants. Phase two consisted of broader, contextual triangulation, in which the results from individual participants were combined and linked to more general data emerging from the qualitative data. After study completion one FGD with purposively selected study participants was conducted to probe further into ring expulsions.

### Adherence and outcome measures

The level of adherence was (semi-)quantified in multiple ways. Adherence to the ring was based on observed product use at the visits and self-reported use. Observed product use was done during the scheduled pelvic exam by a physician and the following questions was answered on the CRF “Was the ring in place at the start of the visit?” For the self-reported use, we first considered the answer to the following question on the CRF and IAQ “Since your last regularly scheduled visit, how many times was the vaginal ring out of your vagina?” The response could be a number or a separate answer stating “the ring was never out of the vagina”. The latter (the ring was never out) was defined as *perfect* adherence (Table 1). If the former (the answer was a number of times the ring was out), questions were asked about the last (most recent) time the ring was out first, followed by questions about the other times that the ring was out. These included questions about how the ring came out or was removed, the duration that the ring was out, and whether the ring was reinserted. These events were coded as high, mid-high, mid-low, low, and non-adherence depending on the duration (in hours) that the ring was out of the vagina since the duration the ring is out of the vagina corresponds to levels of pregnancy risk (40–42). According to the NuvaRing® user instructions, a woman is considered to be at risk of pregnancy if the ring has been out for more than three hours, therefore in the categories mid-low, low, and non-adherence, protection from pregnancy may be reduced if a (new) ring is inserted more than 3 h after expulsion or removal (40). As an example, a participant was assigned to the mid-low adherence category if she reported that the ring was out of the vagina for a duration between 3 and 12 h since the previous visit. If the ring had been out of the vagina multiple times since the last regularly scheduled visit, the longest duration out of the vagina was considered to categorize self-reported adherence.

**Table 1:** Overview of adherence measurements.

Adherence categories			
Monthly ring adherence	Self-reported ring adherence by study group and by visit based on duration (in hours) that the ring was out of the vagina.	Perfect adherence	Ring never out
		High adherence	Ring out < 1 h
		Mid-high Adherence	Ring out between 1 and 3 h
		Mid-low Adherence	Ring out between 3 and 12 h
		Low adherence	Ring out between 12 and 24 h
		Non-adherent	Ring out > 24 H
Monthly ring adherence (montgomery)	Self-reported ring adherence by study group and by visit based on the proportion (percentage) of whole or partial days (12 h or more) that the participants wore the ring by dividing the number of days that the ring was worn by the number of days that the ring should have been worn since the last regular scheduled visit.	Perfect adherence	Ring inserted 100% of 21 days
		Ring inserted > or equal to 80% of 21 days	
		Ring inserted < 80% of 21 days	
		Ring inserted < 50% of 21 days	
Cumulative ring adherence	For the cumulative adherence categorisation, this was done by adding all durations the ring was out during the study to arrive at an overall duration that the ring was out (in hours).		
Cumulative ring adherence (montgomery)	For the Montgomery cumulative adherence categorisation, this was done by dividing the number of days that the ring was worn across the duration of the study by the number of days that the ring should have been worn during the study.		

This type of categorization represents the rigorousness and strict follow-up of a clinical trial setting. It is less useful for assessing real-world contraceptive effectiveness or to inform public health counselling and messaging. Multiple handbooks about family planning state that levels of protection are not affected if the ring was out of the vagina for more than 48 h or less from week 1 through week 3 (40, 43–45). The study team therefore also categorized self-reported adherence data for each visit based on the percentage of whole or partial days that participants wore the ring since the last regularly scheduled visit, as developed by Montgomery et al. (41). This was done by dividing the number of days or partial days (12 h or more) that participants wore the ring by the number of days that the ring should have been worn since the previous visit (21 days). For example, a participant was classified as “80% adherent” if she reported that the ring was in the vagina for 80% of the days since the previous visit. This categorization gives a more appropriate insight into levels of self-reported adherence that matter for behaviour change interventions and are required to achieve contraceptive coverage on a population level. In addition to the measures of self-reported adherence assessed at each visit for the period between the previous and the current visit, overall cumulative ring adherence was calculated across all visits.

Cumulative adherence was added as a measurement to better understand self-reported adherence across a longer period of time (several menstrual cycles) as contraceptives are typically used for a long duration. Data suggests that some contraceptive methods (including the ring) are easier to use the longer they are used as sense of comfort, familiarity etc. increase and initial worries decrease, additionally, publications support findings that familiarity with a delivery method is correlated to preference (29). Cumulative adherence was calculated by adding all durations that the ring was out during the study to arrive at an overall duration that the ring was out (in hours). For the Montgomery adherence categorisation, this was done by dividing the number of days that the ring was worn across the duration of the study by the number of days that the ring should have been worn during the study. To evaluate the potential risk of pregnancy, women were asked whether they had had intercourse during the time that the ring was out of the vagina. Of note, these classifications make no distinction between expulsions and removals.

The self-rating adherence scale was a horizontal line of 10 cm on a piece of paper (Figure 1). Women were asked: “*On a scale from 0 to 10 (0 being not adherent, 10 being perfectly adherent) how would you rate your adherence to the use of the contraceptive vaginal ring since your last visit?*” and to draw a vertical line at the point reflecting their adherence perception. The distance from the left endpoint to the mark was measured in cm.

### Data analysis

The quantitative data were analysed using R, version 4.1.1., and are presented in contingency tables (frequency for categorical data; median and interquartile range (IQR) for continuous data. Chi-square tests were used to compare categorical variables between study groups. The FGD and IDIs were audio-recorded, transcribed in Kinyarwanda, summarised into English, verified and uploaded into Nvivo 10. The open ended questions, the FGD and the IDIs were analysed using a deductive, content-analytical approach using a codebook based on categories related to the quantitative adherence measures. Coded data was summarised by key topics and presented to support and illustrate the quantitative findings.

### Approvals

The study was approved by the Rwandan Ministry of Health and the Rwandan National Ethics Committee (approval number 481/RNEC/2013), the institutional review board of the Institute of Tropical Medicine (ITM) in Antwerp (approval number 864/13), the ethics committee of the University Hospital in Antwerp, Belgium (approval number 13/7/85) and the University of Liverpool in Liverpool, UK (approval number RETG000639IREC). The study was carried out according to the principles stated in the Declaration of Helsinki, all applicable national and international regulations, and the International Conference on Harmonization and World Health Organization Good Clinical Practice guidelines.

## Results

The study team collected quantitative data for all 120 enrolled participants. One participant was lost to follow up after enrolment but the team managed to reconnect with her and complete an end of study visit. The study team collected qualitative data for 104 of the 120 participants in at least one IDI and/or FGD.

### Baseline characteristics of the study population

The baseline characteristics were well-balanced between randomisation groups (Table 2; see also (19). The median age of the enrolled women was 28, 57.5% had a primary school or higher level of education, and 59.2% earned their own income. Most women were married (61.0%) or living with a steady partner (26.7%). About two thirds of the women (65.8%) had previously used a modern contraceptive method (mostly hormonal) and 38.3% had used a condom during their last sex act.

**Table 2:** Socio-demographic characteristics of all enrolled study participants (detailed per study group).

Baseline characteristics	All participants	Intermittent use	Continuous use
	(*N* = 120)	(*N* = 60)	(*N* = 60)
Age in years: (median; IQR)	28 (26; 31.9)	28 (25.5; 31)	28.5 (26; 32)
Highest level of education: *n* (%)			
No schooling	15 (12.5)	9 (15.0)	6 (10.0)
Primary school not completed	36 (30.0)	15 (25.0)	21 (35.0)
Primary school completed	44 (36.7)	24 (40.0)	20 (33.3)
Secondary school not completed	17 (14.2)	8 (13.3)	9 (15.0)
Secondary school completed	4 (3.3)	2 (3.3)	2 (3.3)
More than secondary school	4 (3.3)	2 (3.3)	2 (3.3)
Age of first intercourse in years: (median; IQR)	18 (15.7–21.1)	18 (15.5–20.8)	18.5 (15.8–21.4)
Marital status/home situation: *n* (%)			
Married	73 (60.8)	37 (61.7)	36 (60.0)
Not married, regular sex partner, living together	32 (26.7)	16 (26.7)	16 (26.7)
Not married, regular sex partner but not living together	15 (12.5)	7 (11.7)	8 (13.3)
Contraception history: *n* (%)			
None	41 (34.2)	19 (31.7)	22 (36.7)
Hormonal	79 (65.8)	41 (68.3)	38 (63.3)
IUD^†^	1 (0.83)	1 (1.7)	0 (0)
Injectables^†^	59 (49.2)	32 (53.3)	27 (45.0)
Pills^†^	29 (24.2)	11 (18.3	18 (30.0)
Condom use at last sex act: *n* (%)	46 (38.3)	23 (38.3)	23 (38.3)
Any vaginal deliveries: *n* (%)	106 (88.3)	55 (91.7)	51 (85.0)
Any C-sections: *n* (%)	17 (14.2)	6 (10.0)	11 (18.3)
Ever anal sex: *n* (%)	1 (0.8)	1 (1.7)	0 (0)
Ever sex during menses: *n* (%)	18 (15.0)	5 (8.3)	13 (21.7)
Ever transactional sex: *n* (%)	9 (7.5)	5 (8.3)	4 (6.7)
Worried about getting HIV: *n* (%)			
Very worried	25 (20.8)	11 (18.3)	14 (23.3)
A little worried	41 (34.2)	21 (35.0)	20 (33.3)
Income			
Own income: *n* (%)	71 (59.2)	37 (61.6)	34 (56.7)
Average weekly income in Rwandese Francs	20,685	16.828 RwF	24.413 RwF

### Categorising self-reported adherence

#### Monthly adherence

**Figure 2:**
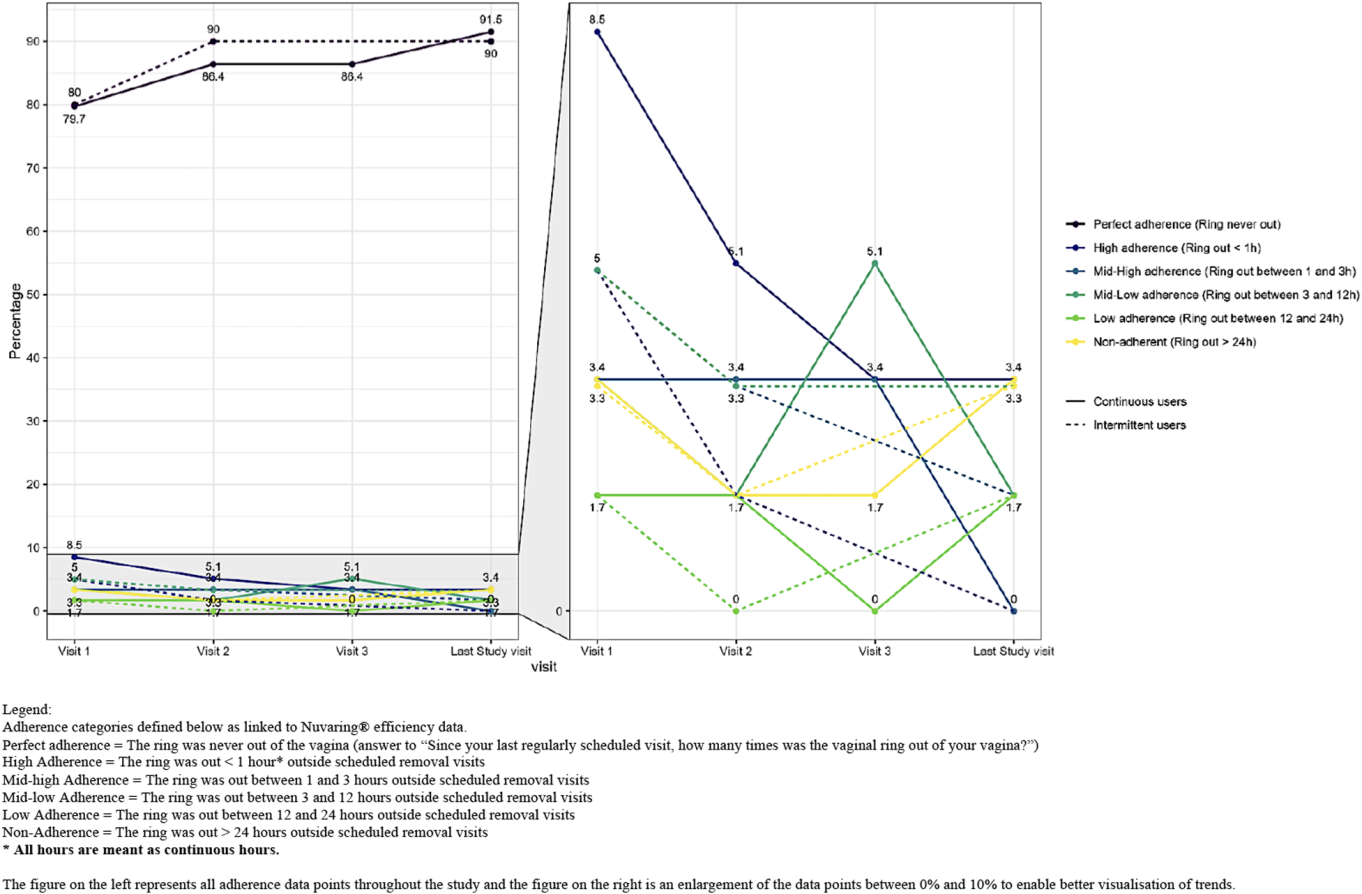
Self-reported ring adherence by group and by visit based on duration (in hours) that the ring was out of the vagina. Legend: Adherence categories defined below as linked to Nuvaring® efficiency data. Perfect adherence = The ring was never out of the vagina (answer to “Since your last regularly scheduled visit, how many times was the vaginal ring out of your vagina?”). High adherence = The ring was out < 1 h* outside scheduled removal visits. Mid-high adherence = The ring was out between 1 and 3 h outside scheduled removal visits. Mid-low adherence = The ring was out between 3 and 12 h outside scheduled removal visits. Low adherence = The ring was out between 12 and 24 h outside scheduled removal visits. Non-adherence = The ring was out > 24 h outside scheduled removal visits. * All hours are meant as continuous hours. The figure on the left represents all adherence data points throughout the study and the figure on the right is an enlargement of the data points between 0% and 10% to enable better visualisation of trends.

Self-reported adherence categorised by the duration (in hours) the ring was out of the vagina was high at every regular scheduled visit in both study groups (Figure 2 and Supplementary S1). Eighty percent of the intermittent users and 77.9% of the continuous users reported perfect adherence at the first scheduled study visit and this increased to 90% for both groups at the last scheduled study visit. Ten percent of the intermittent users and 13.6% of the continuous users were categorised as having high or mid-high adherence (which means they were still protected from pregnancy) at the first study visit and this decreased to 1.7% and 3.3%, respectively at the last study visit because perfect adherence increased. Of the seven intermittent users reporting high or mid-high adherence at the first scheduled study visit, six became and remained perfect adherers during the study. One woman reported perfect adherence at the second study visit but mid-high adherence at the last study visit due to ring removal because of discomfort. Of the eight continuous users reporting high or mid-high adherence at the first study visit, four became perfect adherers during the study, one was perfect adherent except for being high adherent at the third study visit. For the other three; one remained a high adherer at the second study visit and two reported respectively mid-low and low adherence at the second study visit. At the third study visit those three became perfect adherers and all remained perfect adherers till the end of the study.

**Figure 3:**
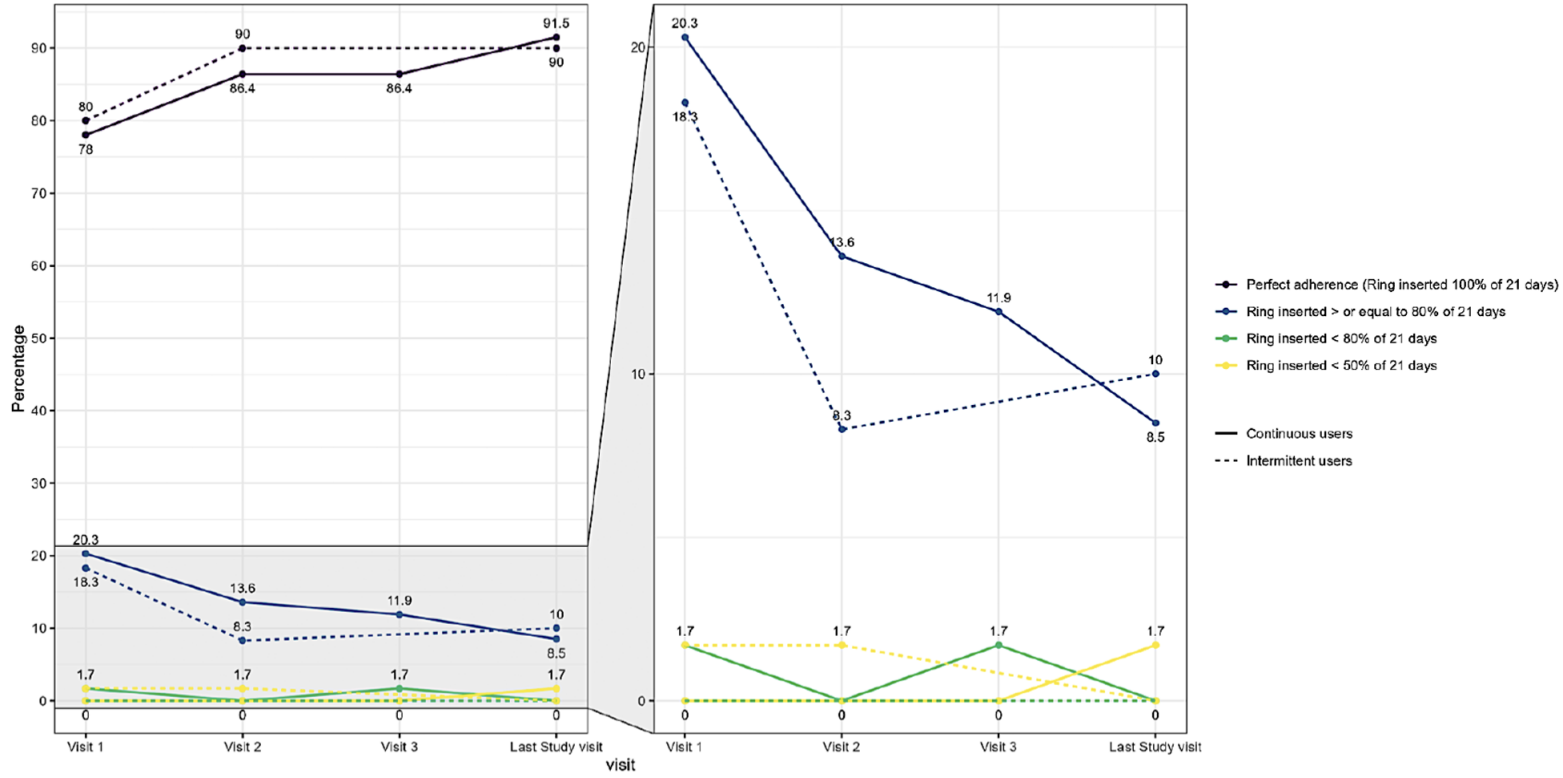
Self-reported ring adherence by study group and by visit based on proportion of whole or partial days that participants wore the ring. Legend: Adherence categories defined below as linked to Nuvaring® efficiency data. Perfect adherence = The ring was never out of the vagina (answer to “Since your last regularly scheduled visit, how many times was the vaginal ring out of your vagina?”). High adherence = The ring was inserted > or equal to 80% of 21 days. Mid adherence = The ring was inserted < 80% of 21 days. Low adherence = The ring was inserted < 50% of 21 days. The figure on the left represents all adherence data points throughout the study and the figure on the right is an enlargement of the data points between 0% and 20% to enable better visualisation of trends.

The self-reported adherence categorised by the percentage of whole or partial days participants wore the ring was also high at each visit in both study groups (Figure 3 and Supplementary S2). The majority of women in both groups were perfect adherers as described before. Using this adherence definition, 20% of the intermittent users and 18.6% of the continuous users were categorised as having good adherence (ring inserted for at least 80% of the expected 21 days) at the first study visit and this number decreased during the study to 6.7% at the last study visit as perfect adherence increased. None of these percentages were statically significant between the study groups. Adherence according to the self-rating scale was also high. In the intermittent use group, the median adherence value was 9.6 (visit 1), 9.7 (visit 2) and 9.7 (last study visit). In the continuous use group, the adherence data was 9.4 (visit 1), 9.4 (visit 2), 9.7 (visit 3) and 9.6 for the last study visit.

#### Cumulative adherence

Of the 120 participants enrolled, 119 had self-reported adherence data available for each of the three/four scheduled study visits and were included in the assessment of cumulative adherence. The percentage of women with perfect cumulative adherence was non-significantly higher in the intermittent use group (61.7%) than in the continuous use group (54.3%) (Table 3). However, 23.3% of the intermittent users and 23.8% of the continuous users were at risk of getting pregnant at some point during the study period based on their reported adherence levels based on the number of hours that the ring was out of the vagina. The percentages of women with 80%–100% cumulative adherence based on the percentage of days that the ring was inserted were 93.4% and 96.7% for intermittent vs. continuous use, and only 6.6% and 3.3%, respectively, were considered non-adherent. None of the women became pregnant during the study. For the last study visit we also collected self-rating adherence data on the ballot box questionnaire for all participants. This was done anonymously so the data represents both study groups together. For the question: “*Over the course of the study, was the ring out for more than three hours at any time (not considering the clinic visits or ring free periods*)?” a total of 33 women ticked “YES”. This amounts to 27.5% of the participants, similar to the cumulative adherence data based on hours that the ring was out of the vagina.

**Table 3:** Cumulative ring adherence per group.

Cumulative number of hours during the study^a^	Participants		Cumulative number of days during the study	Participants	
Study group	Inter. use (*n* = 60)	Cont. use (*n* = 60)	Study Group	Inter. Use (*n* = 60)	Cont. use (*n* = 60)
Perfect adherence (Ring never out)	37 (61.7%)	32 (54.3%)	Perfect adherence (ring inserted 100% of days)	37 (61.7%)	32 (53.3%)
High adherence (Ring out < 1 h)	3 (5.0%)	9 (15.3%)	Ring inserted 80–99% of days	19 (31.7%)	26 (43.4%)
Mid-High adherence (Ring out between 1 and 3 h)	6 (10.0%)	4 (6.8%)	Ring inserted 50–79% of days	0	0
Mid-Low adherence (Ring out between 3 and 12 h)	7 (11.7%)	6 (10.2%)			
Low adherence (Ring out between 12 and 24 h)	2 (3.3%)	3 (5.1%)	Non-adherent (ring inserted 0–49% of days)	4^b^ (6.6%)	2^b^ (3.3%)
Non-adherent (Ring out > 24 h)	5^b^ (8.3%)	6^b^ (8.5%)			

^a^ Study duration = 3 × 21 days (63 days) for the continuous use group and 4 × 21 days (84 days) for the intermittent use group.

^b^ 4 unknown for the intermittent use group, 1 unknown for the continuous use group.

#### Removals and expulsions

**Figure 4:**
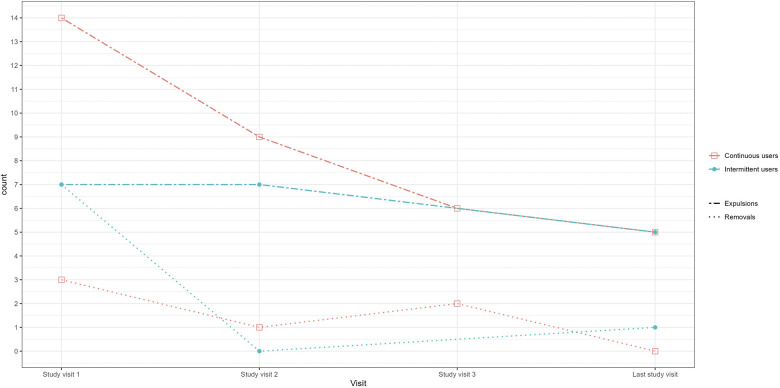
Self-reported ring removals and expulsions by study group and visit. Legend: Count = The number of times the ring was removed or expelled between two study visits. For example for study visit 1, the number of times the ring was removed or expelled since the enrolment visit. For study visit 2, the number of times the ring was removed or expelled since study visit 1, et.

During the course of the study, 23 intermittent users (16.1%) reported 21 ring expulsions and eight ring removals out of a total of 180 ring insertions in that group, and 27 continuous users (17.3%) reported 36 ring expulsions and five ring removals out of a total of 237 ring insertions. Reporting of ring expulsions and removals were highest at the beginning of the trial and declined during the study (Figure 4). The most frequent event associated with vaginal ring expulsion in the intermittent users was defecation and urination, which were associated with 28.6% of expulsions (*n* = 11 and *n* = 14, respectively; Supplementary S3). The most common reason for ring removal was discomfort/pain experienced by the participant or her partner (*n* = 5). Three rings were removed at the request of the male partner. There was no statistical difference between both study groups in terms of percentage of expulsions (*p* = 0.372) or removals (*p* = 0.283).

The intermittent users who reported ring removals or expulsions reported to have re-inserted the ring themselves 12 times (41.4%), and the ring was reinserted by clinic staff twice (6.9%) (Supplementary S3). The ring was rinsed with water 37.9% of the time, no one used soap as instructed. In the continuous user group, the ring was reinserted by the participants themselves 25 times (60.9%) and not reinserted by clinic staff. Half of the time (51.2%) the ring was rinsed with water, no one used soap. Although the majority of women did not engage in intercourse without the ring, one woman in the intermittent use group and nine in the continuous use group had sexual intercourse without the ring but all, except four in the continuous use group, used a condom.

### Perceptions of adherence

When probing about how women defined adherence in our pre-trial IDIs, most of the women put a lot of weight on being compliant with study procedures which included coming on the right day and at the right time for the scheduled appointments.

*P: “Adherence is fulfilling the program you have with someone or to do something that you have planned to do and you do it on time.” (29, married, 2 children)*

*P: “Adherence is like you have an appointment somewhere and you go there.”*

*P: “It is like you respect something, if they give you an appointment, you come on the time they gave you, don't miss the appointment.” (35, married, 1 child)*

This finding was addressed by repeating how the study team defined adherence when subsequent questions were asked during the trial and at study end. On the last study visit we asked the following open-ended question: “*How would you describe your adherence to the ring over the entire study?”* Not a single woman (except the person who was lost to follow-up) stated that they had problems. The woman who was lost to follow-up came back for a last ring visit; she told the team that she stopped using the ring because she had not wanted to disclose her ring use to her partner. Almost half of the participants thought their adherence was perfect (*n* = 54).

*Participant (P): “My adherence over the entire study was perfect, both myself and husband have liked the ring and would like to continue using it.” (29, not legally married, 2 children)*

The representation of the participants as being perfectly adherent was supported by additional insights provided by the IDIs.

*P: “I would give myself 10/10.*

*Moderator (M): “10/10 meaning that you would adhere to all programs, isn't what you told me that participation is? Adhering to all programs?”*

*P: “Yes.” (29, married, 4 children)*

Eventhough the study team clarified our understanding of “adherence” at several time points during the study, we still found that several women stressed the fact that they were responsible in following all study procedures and appointments given (*n* = 51) and had provided accurate information during the study.

*P: “My adherence to the ring over the entire study was perfect, I managed to use the ring correctly as instructed and all information that I have given for the ring are true.” (21, not legally married, 1 child)*

*P: “I have liked the ring from the beginning that’s why I said that it was my responsibility to use it well as instructed and I have made it.” (33, not legally married, 3 children)*

### Triangulated data

A first phase of triangulation where data from different data collection tools were compared showed that 15 intermittent users provided discrepant reports on the CRF vs. the IAQ at 15/180 (8.33%) of all scheduled visits and nine continuous users at 10/240 (4.17%) of scheduled visits (Table 4). One participant reported discrepancies at two different visits. All discrepancies consisted of participants mentioning the ring was not out on the CRF but stating the ring was out (including answering all follow-up questions) on the IAQ. These inconsistencies were resolved internally by the study team based on the assumption that the IAQs provided more accurate information as the forms were less standardized and more extensive with in-depth follow-up questions about ring removals and expulsions. Additionally, more time was allocated to completion of the IAQ which was conducted by the study nurses whereas the CRFs where completed by the study physicians.

According to the IAQ data, 12 women in the intermittent use group and 14 women in the continuous use group (a total of 26 women) reported expulsions and/or removals longer than 3 h. These results indicate that seven women who ticked ‘YES” on the ballot box, did not report the ring being out for more than three hours on the IAQ (Table 4). On the ballot box form, we also asked participants to self-rate their overall adherence throughout the trial on the self-rating scale. The median self-reported adherence value was 9.6 (five missing values). Two women scored themselves below 5, two below 7, three 8.8, and 108 (90%) between 9 and 10.

When we compared these findings (self-rated adherence scale results) to other self-reported adherence data collected through the IAQs and CRFs across study visits, we found 21 participants with inconsistent data of which 9 findings related to inconsistent adherence data and addressed these in the comparison forms. One example is of a participant who stated that she had been perfectly adherent throughout the study. However, she marked her adherence on the self-rating scale as 7.3 (visit 1), 6.2 (visit 2) and 9.8 (last study visit). We asked the participant to explain in her own words about her ring use experience and adherence over the entire trial. She answered:

*P: “I found that there are no negative effects of using the ring, it is easy to wear the ring, I did not feel it during my routine activities, I did not get any discomfort from ring. I respected all instructions & appointments so, no bad experience I got from using ring.” (33, married, 3 children)*

**Table 4:** Overview of discrepant adherence results across entire study duration.

Case report forms (CRF) data stating “NO” but interviewer-administered questionnaire (IAQ) data stating “YES” for removals and expulsions				
Study Visit	Study Group			
	Group A *n* = 180		Group B *n* = 240	
Ring Visit 1				
	4 discrepancies	All follow-up questions are answered in the IAQ	2 discrepancies	All follow-up questions are answered in the IAQ
	1 discrepancy	IAQ mentions flushed down toilet		
	**5**		**2**	
Ring Visit 2				
	2 discrepancies	All follow-up questions are answered in the IAQ	1 discrepancy	In a dirty place
	1 discrepancy	She came to the clinic with the ring	1 discrepancy	In the toilet
	1 discrepancy	IAQ mentions flushed down toilet		
	**4**		**2**	
Ring Visit 3				
	none		2 discrepancies	IAQ mentions flushed down toilet
	**0**		**2**	
Last Ring Visit				
	2 discrepancies	All follow-up questions are answered in the IAQ	4 discrepancies	IAQ mentions flushed down toilet
	4 discrepancies	IAQ mentions flushed down toilet		
	**6**		**4**	
	**15 discrepant results (8.33%)**		**10 discrepant results (4.17%)** *same participant had 2 discrepancies	
**Total 25 discrepant results (5.95%)**				
** **				
IAQ data stating “NO” but ballot box stating “YES” for Expulsions and/or removals longer than 3 h				
Study Visit	Study Group			
	Group A&B *n* = 120			
Last Ring Visit	26 vs. 33			
**Total 7 discrepant results (5.83%)**				

Bold values show the total amount of discrepant results between CRF and IAQ for all study participants across the whole study duration *n*=25 (5.95%). The total amount of discrepant results between IAQ and Ballot box for all study participants across the whole study duration *n*=7 (5.83%).

When we probed further about the marking of the adherence scales, she told the team that she had included adherence with study procedures in her overall adherence assessment, such as the time (of day) that she had completed the diary card and whether she had made mistakes (overwriting) on the forms. This is in line with our prior mention about adherence being understood as adherence with all study procedures despite acknowledging this challenge early on.

Even though the diary card was not considered a data collection tool but rather a support for the participants to minimize recall bias when answering the IAQ at their scheduled visits, the team did revise them and documented any inconsistencies found between the IAQ and the diary card. We probed about this during the comparison form discussions, and participants had reasonable explanations revealing most of the inconsistencies were around a misunderstanding of the diary card completion instructions.

At study completion, the team proceeded with the second phase of the broader triangulation where all available self-reported adherence data was analysed across the entire study. By doing so, the team noticed very similar, almost identical responses given by different participants for some ring expulsions; the ring being flushed down the toilet due to defecation and women coming back to the clinic for a new ring to be inserted. We found potential links between most of these participants, either they were living in the same house or in the same neighbourhood, or had been invited on the same day and hence had spent time together in the waiting area. After deliberation, the team decided to invite those women for a focus group discussion to delicately probe into possible explanations. Not only were the answers too similar but the number of expulsions was exceeding what would have been expected based on the available Nuvaring® leaflet expulsion data. We shared with the women what we had found in the data, the importance of accurate data collection in research, and explained these results were not in line with other findings. We explained the participants were selected due to their ring expulsions experiences and we wanted to better understand what had happened. Women shared that the ring would come out mainly because of either not paying attention or because of illness.

*P: “My ring came out, but I felt that it was coming out, I had diarrhoea, but if I was paying attention, I would save it and get it before it fall down.” (FGD 15feb2014)*

The participants stressed that they thought that the design of the ring was not problematic.

*P: “You cannot blame this ring for anything, it is a nice method of contraception, I give an example of mine, I tried all other FP methods, and I failed due to side effects…” (FGD 15feb2014)*

We shared with the participants that ring expulsion rates in this study were higher than we had anticipated based on data from other studies and we wanted to know if the participants had heard of women coming to the clinic for unnecessary unscheduled visits to obtain a new ring. Whilst most of the discussions centred around “other” women, and the gossip they heard in the waiting area, some women did confess to providing false information and did express their regret about that (although in a general sense not specific to ring expulsions).

*P: “Shame on us, shame on us to give bad information!” (FGD 15Feb2014)*

One of them expressed regret of not having given the correct information by saying:

*P: “Yoooo, shame on us by giving false information! Is it too late, can't you hold a bit to give out the report and we start from zero? So that we will give you the new information? This ring has no problem, it is just us who…” (FGD 15Feb2014) Silence, she looked down, seemed to hide something else she wanted to say.*

We asked all participants who did not reinsert their rings during the study due to flushing it down the toilet or dropping it on a dirty surface/place if they were willing to be part of a FGD to provide more information. For the intermittent use group, seven participants were invited and for the continuous use group, eight participants were invited; total *n* = 15 or 12.6% of study population. We did not invite the participant who was lost to follow-up as she had already explained at her last study visit that she was not completely honest about the reasons for flushing her ring down the toilet. Six participants (40% of invited women) came for the FGD, and we asked these women if they wanted to come back afterwards to discuss some issues further with the team. Four of them came (about 25% of all invited women) and some even brought back the “lost” ring and admitted to having provided false information. We learned from the informal discussions at these extra visits that some women came for unnecessary unscheduled visits to receive transport reimbursement, and not because they had actually lost their ring, or because they wanted additional rings for future use or to give to others.

## Discussion

Self-reported ring adherence at each study visit was high across the two study groups with around 80% of participants reporting perfect adherence at the first study visit increasing to around 90% at the last study visit. These findings are in line with previous studies showing very similar data on self-reported adherence to the Nuvaring® with adherence ranging from 80% to 91% as well as more recent data of 92% self-reported adherence to novel multidrug vaginal rings (46–48). Of interest is that self-reported adherence was not significantly different between the two study groups indicating that continuous users did not seem to have more adherence challenges. Unfortunately the reliability of self reported data on contraceptive adherence is often questioned although no golden standard is currently available (49, 50). Literature on self-reported adherence measurements indicates general over-reporting of adherence due to social desirability bias and overreporting is also linked to socio-demographic variables such as age and social determinants such as relationship dynamics (26, 47, 51–53). As our study findings rely on participant self-report, similar limitations should be highlighted in terms of social desirability and recall bias. Additionally, researchers assume that clinical trial patterns differ from “real world” behaviours and that adherence in clinical trials usually is as good or greater than “real life” adherence (16, 18, 23, 54, 55).

Based on prior research and literature on over-reporting, the team tried to minimize incorrect reporting by developing a robust study design including triangulation, appropriate counselling, and support tools like diary cards (56–58). Our triangulated study results suggest a low discrepancy rate overall. However, the team did find some overreporting of expulsions/removals fuelled by socio-economic motivators, and underreporting of adherence due to the widely held perception that adherence includes adherence to all study procedures. However, both of these led to underreporting of ring use adherence as opposed to overreporting. Based on the collected data and our extensive triangulation, over-reporting of adherence seems a less important challenge in our study population than previously assumed. Whilst some previous cited publications indicate a tendency to over-report adherence, other studies using adherence biomarkers like Chen et al. (2015) report good correlation between self-report and residual drugs levels in returned vaginal rings (59). Our findings indicate that behaviour and reporting related to adherence are complex and do not always lead to over-reporting of good adherence or “perfect” use in a clinical trial setting.

Perfect self-reported cumulative adherence across the entire study period was 61.7% for intermittent users and 54.3% for continuous users. These results show that whilst a large group of women were perfectly adherent at each visit, not all of these women were perfectly adherent for each single visit throughout the study. This in itself is not unexpected as potential for expulsions and/or removals will increase with longer duration of contraceptive use but it does indicate that eventhough most published data indicates that adherence seems to increase with ring use, maintaining “perfect use” over a longer period of time is challenging in our population (27, 47)

Interestingly, even with periods of the ring being out, participants still represented themselves as being perfectly adherent. As opposed to our findings on underreporting of perfect adherence, this finding of perceived good adherence is in line with a vast majority of other publications echoing the idea of being a responsible, good study participant, highlighting the participant's moral integrity framed within determining social and contextual factors (24, 54, 58–60). In his publication “Adherence and the Lie in a HIV Prevention Clinical Trial”, Stadler et al. argued for a more nuanced perception of perceiving non-adherent participants not solely as liars or purposely deceiving the study team but as actors in something far more complex, impacted by the context of structural factors like poverty, social inequalities and limited or inequitable health care systems (54, 60, 61). Equally important to note, is that imperfect adherence does not always equal increased risk of pregnancy.

Whilst the Nuvaring® manufacturer's instructions indicate a higher risk of pregnancy if the ring is out of the vagina for a single time for three hours or more, other user instructions such as Planned Parenthood, WHO or the Center for Disease Control refer to increased risk after 48 h of the ring being out or in case of the Sexual Health Victoria website 24 h (during the first three weeks of use) (40, 43–45). Based on the Nuvaring® leaflet instructions, our data indicates that 21.6% of the intermittent users and 23.8% of the continuous users were at risk of getting pregnant during the study period. None of our participants became pregnant during the study which could indicate the Nuvaring® manufacturers user instructions might be conservative estimates. However only four women in the continuous use group had completely unprotected sexual intercourse without the ring and without a condom. These low numbers might be partly explained by the counselling, testing and treatment, and free condoms participants received in the study. In real word settings, especially for women in stable relationships, condom use might differ (62).

Finally, we experienced that throughout the study the nurses developed a different rapport and a deeper relationship with the study participants as compared to the study physicians. This was highlighted on several occasions where the participants confided to the study nurses about challenges they were facing in daily life which were not asked about in the context of our study as well as the nurses trying to help the participants on different occasions with problems outside of the research centre's remit. The impact of staff characteristics and attitudes on honest reporting has been mentioned by other researchers like Van der Straten et al. and Montgomery et al. who stated that: “*There was no clear consensus about many of these issues, though participants often preferred female staff and those who were “nice” and not rushed or harsh*” (16, 54). Interestingly Stadler's research also showed that the deeper relationship developed with the study nurses led to increased desirability bias where the participants did not want to disappoint the study nurses (61). When reviewing the triangulated self-reported adherence data, inconsistencies were found between the CRF, the IAQ (the self-rating adherence VAS) and the diary cards. Although some researchers like Toley et al. state that responses collected at the last study visit are more honest or accurate (42), there is no general consensus, and more recently researchers like Mensch et al. have found that disclosure of non-adherence did not increase at last visit compared to adherence reporting throughout the study (51). Our study team's view was that whether non-adherence reporting would increase or not, they did not want to jeopardize the rapport developed with the participants or risk losing their trust, and hence avoiding any change in reporting due to an increase in desirability bias or loss of trust. Therefore, they did not address inconsistencies whilst the study was ongoing but they addressed them at the last study visit using individual comparison forms once all data collection was completed.

When asked about internal discrepancies between the IAQ answers including open-ended questions and the self-rating adherence VAS, it became clear that the concept of adherence had different meanings to different participants. Even though the study team had piloted the concept of adherence during data collection tool development, the participants shared different conceptualizations of “consistent use” and “adherence” as has been reported by several other researchers including Montgomery et al. (26). Additionally, the same adherence levels were assessed differently by different participants as also noted by Mensch et al. reporting that in HIV trials, “*the question asking participants to rate their ability to keep the vaginal ring inserted as instructed is subject to varying interpretations such that the same level of adherence might be assessed differently by different participant.*” (55). The study team decided to report the data as was shared by the participants during the study. Development of adherence biomarkers or other biological/surrogate markers has progressed, and whilst they provide crucial data on product (non)use, all remain user dependent to a degree and do not asses or clarify adherence and behaviour patterns (17, 59). Context specific, simple adherence measures and data collection procedures should remain priorities because they provide a deeper understanding of context-driven behaviour that shapes adherence patterns and challenges (17, 49, 56–58, 61–62). Our findings highlight the importance of in-depth qualitative work in parallel with the development of more accurate biomarkers for adherence measurements.

## Conclusion

Self-reported adherence to the Nuvaring® over the entire study duration was high in both the intermittent and continuous first-time vaginal ring users. Rwandan policy makers should consider the Nuvaring® as a valuable addition to the current family planning package. In this manuscript, we highlight the importance of having a well-designed study and we demonstrate that using mixed methods and triangulation in particular allows for better understanding of adherence and the possibility of addressing inconsistencies in an appropriate and relevant manner. Despite advances in the development of biomarkers and other biological/surrogate markers in contraception and HIV prevention research, self-reported adherence measures will always play a crucial role. Research into context specific, simple adherence measures remains important to the ongoing development of the pipeline of new female-initiated drug-delivery methods. Future ring adherence studies might opt for a more pragmatic design including a strong qualitative component with our data categorisation and triangulation approach to better account for real-world non-adherence.

## Limitations

This manuscript focusses on adherence measurements and adherence data captured in the Ring Plus study. The importance of broader socio-cultural and structural factors that influence adherence, eventhough very important, are outside of the scope of this manuscript. Our study findings rely on participant self-report and as such are prone to social desirability and recall bias. The study team mitigated this through a robust study design but it is not possible to eliminate all biases completely and should therefore be mentioned as a limitation.

## Data Availability

The raw data supporting the conclusions of this article will be made available by the authors, without undue reservation.

## Ring plus study group

Jozefien Buyze PhD, Vicky Cuylaerts, Gloria Gahindu, Liselotte Hardy, Vicky Jespers PhD, Joris Menten, Viateur Musengamana, Jean Claude Ndagijimana MD, Raffaella Ravinetto, Celine Schurmans, and Harry Van Loen. The lead author for this group is Liselotte Hardy (lhardy@itg.be).
