# Supplementary material for: High adherence to intermittent and continuous use of a contraceptive vaginal ring among women in a randomized controlled trial in Kigali, Rwanda
Source: Front Glob Womens Health. 2024 Apr 11;5:1278981. doi: 10.3389/fgwh.2024.1278981 (PMC11047128; doi:10.3389/fgwh.2024.1278981)
Supplement: Supplementary file 1 [file Datasheet1.pdf]

Supplements

Supplement 1: Self-reported ring adherence by group and by visit based on duration (in hours) that the ring was out of the vagina.

	Visit 1		Visit 2		Visit 3	Last Study Visit	
**Study Group**	Inter. use (n=60)	Cont. use (n=59)*	Inter. use (n=60)	Cont. use (n=59)	Cont. use (n=59)	Inter. use (n=60)	Cont. use (n=60)
Perfect adherence (Ring never out)	48 (80%)	46 (77,9%)	54 (90%)	51 (86,4%)	51 (86,4%)	54 (90%)	54 (90%)
High adherence (Ring out < 1 h)	3 (5%)	5 (8,5%)	1 (1,7%)	3 (5,1%)	2 (3,4%)	0 (0%)	2 (3,3%)
Mid-High adherence (Ring out between 1 and 3 h)	3 (5%)	3 (5.1%)	2 (3,3%)	2 (3,4%)	2 (3,4%)	1 (1,7%)	0 (0%)
Mid-Low adherence (Ring out between 3 and 12 h)	3 (5%)	2 (3,4%)	2 (3,3%)	1 (1,7%)	3 (5,1%)	2 (3,3%)	1 (1,7%)
Low adherence (Ring out between 12 and 24 h)	1 (1,7%)	1 (1,7%)	0 (0%)	1 (1,7%)	0 (0%)	1 (1,7%)	1 (1,7%)
Non-adherent (Ring out > 24 h)	2 (3.3%) 1 day and unk	2 (3,4%) 4 and 5 days	1 (1,7%) unk	1 (1,7%) 2 days	1 (1,7%) 1,5 days	2 (3,3%) unk	2 (1,7%) unk and 37 days

***One woman from the continuous use group only came for enrolment and last ring visit but missed her regular visit 1,2 and 3.**

If the ring was out multiple times, the longest duration was taken into consideration to calculate adherence.

Supplement 2: Self-reported ring adherence by study group and by visit based on proportion of whole or partial days that participants wore the ring.

	**Visit 1**		**Visit 2**		**Visit 3**	**Last Study visit**	
Study Group	Inter. use (n=60)	Cont. use (n=59)	Inter. use (n=60)	Cont. use (n=59)	Cont. use (n=59)	Inter. use (n=60)	Cont. use (n=60)
Perfect adherence (Ring inserted 100% of 21 days)	48 (80%)	46 (77.9%)	54 (90%)	51 (86,4%)	51 (86,4%)	54 (90%)	54 (90%)
Ring inserted > or equal to 80% of 21 days	11 (20%)	11 (18.6%)	5 (8.3%)	8 (14%)	8 (14%)	4 (6.7%)	4 (6.7%)
Ring inserted < 80% of 21 days	0	1 (1,7%)	0	0	0	0	0
Ring inserted < 50% of 21 days	1 (1,7%) unk	0	1 (1,7%) unk	0	0	2 (3.3%) unk	2 (3.3%) unk and 37 days

Supplement 3: Reasons for ring removals and expulsions by study group and visit.

	Visit 1		Visit 2		Visit 3	Last study visit		All visits			
Study group	Int	Cont	Int	Cont	Cont	Int	Cont	Int	Cont	Total	P-value
**Reasons for expulsion**											
During defecation	1	0	2	1	1	3	3	6	5	11	
During urination	2	2	2	4	2	2	0	6	8	14	
During sex	1	6	0	4	1	0	1	1	12	13	
After sex	2	3	2	0	0	0	1	4	4	8	
During/after physical activity	0	1	0	0	0	0	0	0	1	1	
Unknown	3	4	0	0	2	0	0	5	4	9	
**Total expulsions**	**9**	**16**	**7**	**9**	**6**	**5**	**5**	**21**	**36**	**57**	
**% of total ring insertions***	**5.0**	**6.8**	**3.9**	**3.8**	**2.5**	**2.1**	**2.1**	**11.7**	**15.2**	**13.7**	**0.372**
**Reasons for removal**											
Causing discomfort/pain	1	2	0	0	1	1	0	2	3	5	
Felt it was not in place	3	0	0	0	0	0	0	3	0	3	
Partner told me to remove it	1	0	0	1	0	0	0	1	1	2	
Other: Show to partner	0	0	0	0	1	0	0	0	1	1	
Other: Unknown	2	0	0	0	0	0	0	2	0	2	
**Total removals**	**7**	**2**	**0**	**1**	**2**	**1**	**0**	**8**	**5**	**13**	
**% of total ring insertions***	**3.9**	**0.8**	**0**	**0.4**	**0.8**	**0.6**	**0**	**4.4**	**2.1**	**3.2**	**0.283**
**Action taken after the ring was out**											
Reinserted herself	9	11	2	6	6	1	2	12	25	37	
Washed with water	8	9	2	6	5	1	1	11	21	32	
Washed with soap	0	0	0	0	0	0	0	0	0	0	
Brought back to the clinic	2	0	2	0	0	1	0	5	0	5	
Reinserted in the clinic	1	0	0	0	0	1	0	2	0	2	
Flushed down the toilet	1	0	1	0	2	4	4	6	6	11	
Dropped in a dirty place	0	0	1	2	0	0	0	1	2	3	
Ring lost	0	1	0	0	0	0	0	0	1	1	
Sex while ring out with condom	1	1	0	2	1	0	1	1	5	6	
Sex while ring out without condom	0	3	0	1	0	0	0	0	4	4	
**% correct actions of total expulsions and removals****	**62.5**	**61.1**	**28.6**	**60**	**75**	**33.3**	**40**	**48.3**	**61**	**55.7**	**0.481**

* Total number of ring insertions was 180 for the intermittent group (n=60) and 237 for the continuous group (n=60 but one woman only had 1 insertion).

** Defined as correct reinsertions either by the participant herself or at the clinic.
