# Supplementary material for: High adherence to intermittent and continuous use of a contraceptive vaginal ring among women in a randomized controlled trial in Kigali, Rwanda
Source: Front Glob Womens Health. 2024 Apr 11;5:1278981. doi: 10.3389/fgwh.2024.1278981 (PMC11047128; doi:10.3389/fgwh.2024.1278981)
Supplement: Supplementary file 2 [file Datasheet2.docx]

**Appendix**

**Appendix 1:** Diary card (English)
